# The Urea Index as a Kidney Test

**Published:** 1918-09

**Authors:** 


**The Urea Index as a Test for Kidney Function in a War Hospital,** By Reginald Fitz, M. R. C. *Journal of the American Medical Association.* June 8, 1918.

Before each test the patient was given from 150 to 200 cc. of water, to insure a free flow of urine. One half hour later, in order to start with the bladder empty, he voided. The time of voiding was recorded to within one minute. About 36 minutes later, at least 3 cc. of blood were withdrawn from an arm vein into a dry tube containing a few miligrams of potassium oxalate to prevent clotting. At the end of 72 minutes from the first voiding, the bladder was again emptied, the urine carefully measured to within 1 cc., and used for analysis. The patient took no food or water during this period. Otherwise no restrictions were placed on him. The 72 minutes period was merely for convenience, as being one-twentieth of 24 hours.

The permanent preparation of urea described by Van Slyke and Cullen was used, and determinations were carried out in the manner described by them, with a few necessary modifications. Three analyses are necessary: blood urea, urine urea, and urine ammonia. The acid used and alkali used in titration are fiftieth normal and hundredth normal alkali and fiftieth normal acid. To carry out the test the apparatus included burets for hundredth normal and fiftieth normal alkali, an aspirating set, and the few necessary reagents and the glassware.

The cases which have been observed are sufficient to show that the urea index test for renal function can be applied satisfactorily in a tent hospital with little if any laboratory facilities. While this procedure is never troublesome where asepsis is certain, under field conditions it is preferable to use intramuscular or intravenous treatment as little as possible.

On the whole, it becomes evident from the cases reported that the urea index is a feasible test for kidney function in a war hospital without particular laboratory facilities. As Mc Lean has said, the method is applicable in a wide range of cases, easy of performance, and gives results that prove of practical value in the recognition, prognosis and treatment of certain conditions associated with impairment of renal function.

